# Automated diagnosis of schizophrenia based on spatial–temporal residual graph convolutional network

**DOI:** 10.1186/s12938-024-01250-y

**Published:** 2024-06-17

**Authors:** Xinyi Xu, Geng Zhu, Bin Li, Ping Lin, Xiaoou Li, Zhen Wang

**Affiliations:** 1 College of Medical Instruments, Shanghai University of Medicine and Health Sciences, Shanghai, China; 2 Shanghai Yangpu Mental Health Center, Shanghai, China

**Keywords:** Schizophrenia, EEG, Temporal frequency characteristics, Spatial frequency characteristics, Graph neural network

## Abstract

**Background:**

Schizophrenia (SZ), a psychiatric disorder for which there is no precise diagnosis, has had a serious impact on the quality of human life and social activities for many years. Therefore, an advanced approach for accurate treatment is required.

**New method:**

In this study, we provide a classification approach for SZ patients based on a spatial–temporal residual graph convolutional neural network (STRGCN). The model primarily collects spatial frequency features and temporal frequency features by spatial graph convolution and single-channel temporal convolution, respectively, and blends them both for the classification learning, in contrast to traditional approaches that only evaluate temporal frequency information in EEG and disregard spatial frequency features across brain regions.

**Results:**

We conducted extensive experiments on the publicly available dataset Zenodo and our own collected dataset. The classification accuracy of the two datasets on our proposed method reached 96.32% and 85.44%, respectively. In the experiment, the dataset using delta has the best classification performance in the sub-bands.

**Comparison with existing methods:**

Other methods mainly rely on deep learning models dominated by convolutional neural networks and long and short time memory networks, lacking exploration of the functional connections between channels. In contrast, the present method can treat the EEG signal as a graph and integrate and analyze the temporal frequency and spatial frequency features in the EEG signal.

**Conclusion:**

We provide an approach to not only performs better than other classic machine learning and deep learning algorithms on the dataset we used in diagnosing schizophrenia, but also understand the effects of schizophrenia on brain network features.

## Background

Schizophrenia (SZ) is a chronic mental disorder characterized by delusions, hallucinations, disorganized speech, and other psychosocial problems [1]. It has adverse effects on patients such as mentality, living skills, occupational and educational performances [2]. Even though the initial symptomatic response is so mild that it can be easily controlled, the consequences of the disease can deteriorate rapidly and become irreversible over time. It is important to recognize the disease as early as possible and administer medication in a timely manner, as a result the approach to diagnose SZ more effectively and accurately is highly demanded.

Generally, the detection of SZ relies on conducting interviews and observing behavioral signs such as hallucinations, functional decline, and disorganized speech [3–5]. However, these methods require a great deal of time and involve tedious steps on the part of a specialized psychiatrist. In the past, researchers devised several methods to assist physicians in diagnosing schizophrenic patients, including magnetic resonance imaging and computed tomography. However, these methods require expensive and bulky equipment and long hours of specialized training [2, 6–10]. Electroencephalography (EEG) signals have been widely used in studies to interpret brain activity and diagnose psychiatric disorders such as depression [11], epileptic seizures [12, 13], autism [14], Parkinson’s disease [15], and Alzheimer’s disease [16], among others. In the field of studying psychiatric disorders, EEG has become the preferred method of detection for SZ due to the portability of the device, non-invasive acquisition, low cost and high temporal and spatial resolution of the acquired signals.

There are two main development areas for the detection of SZ based on EEG. Firstly, statistically topological brain function characteristics. To explain the underlying abnormalities in patients diagnosed with SZ, Shim et al. used three groups of parameters which are 124 sensor-level parameters, 314 source-level parameters, and a combination of both [17]. Bougou et al. focused on the delta and theta bands of EEG signals. They used an abundance of connectivity measures: cross correlation, quadratic magnitude coherence, imaginary part of quadratic magnitude coherence, phase-locked value, phase locked index, p-index, transfer entropy, mutual information, granger causality, partial directed coherence, and directed transfer function [18]. Goshvarpour et al. used nonlinear features, including complexity, Higuchi fractal dimension, and Lyapunov exponents to diagnose SZ subjects with a high precision [19]. Akbari et al. relied on phase space dynamics (PSD) excavation graphical features in EEG signals [20]. Baygin et al. proposed a model for the automatic detection of SZ based on Collatz conjectures using EEG [21]. According to the study of the EEG classification task conducted by Wang Gang et al., Granger causality and SHAP are efficient approaches for measuring pertinent connections in SZ patients [22, 23]. Secondly, data-driven machine learning or deep learning methods with various input features for classification. Some researchers have proven that support vector machine (SVM), linear discriminant analysis (LDA) and k-nearest neighbor (KNN) classification and artificial neural network can help demonstrate the validity of input topographic or dynamic patterns or features, such as Pearson correlation coefficient, relaxed local neighbor difference pattern, continues wavelet transform, discrete wavelet transform, fast Fourier transform, moving averages, and phase lag index [3, 5, 24–29].

Deep learning has been shown to perform exceptionally well among these machine learning techniques in terms of classification accuracy. Specifically, the convolutional neural network (CNN) has emerged as a leading deep learning architecture for processing data in Euclidean space, surpassing the aforementioned machine learning techniques in terms of classification accuracy.

Given the complexity of EEG signals in the spatial and temporal domain, it has been challenging to use deep learning techniques to extract abstract geometric characteristics for improved generalization. The dataset is non-Euclidean because the channels in the structure–function connection network of the EEG are discrete and spatially discontinuous in space. Each EEG channel can be described as a node. The nodes communicate with one another across channels. The cross-channel topologically related EEG features can alternatively be learned using geometric graph-based deep learning techniques. By combining node-specific sequential features and cross-nodes topologically associative features in the graph domain, graph convolutional neural networks (GCN) have been created specifically to handle highly multirelational graph data under the framework of graph theory [30]. In recent years, GCNs have been applied in the diagnoses of various brain disorders, such as children autism spectrum disorder evaluation [31], detection of epileptic [32, 33], seizure prediction [34], epilepsy classification [35], and Alzheimer classification [36]. To the best of our knowledge, there are no SZ diagnostic approaches based on GCN-related models.

The aim of this paper is to develop a deep learning model for analyzing spatial–temporal–frequency 3D features based on spatial–temporal residual graph convolutional neural network (STRGCN). An adjacency matrix based on the wavelet coherence (WC) construct was tested by recording EEG data from SZ patients and healthy controls. STRGCN is able to jointly utilize cross-channel topological connectivity features and channel-specific temporal features. The experimental results show that comprehensively analyzing the temporal frequency and spatial frequency information in EEG can more comprehensively probe the electrophysiological features in the brain.

The main contributions of this paper are as follows:

1. Development of STRGCN to classify SZ from health control (HC).
2. Uncovering schizophrenic patient specificity from multiple dimensions by jointly utilizing cross-channel topological connectivity features and single-channel temporal features.

## Results

The STRGCN method proposed in this study was tested by EEG signal data from public datasets. For comparison, we used convolutional neural networks and long short-term memory network (CNN-LSTM), SVM, LDA, and KNN classification algorithm on the same public datasets. In order to evaluate the significance of time–frequency features and null-frequency features in the identification of schizophrenic patients, the results were analyzed by masking the time–frequency features or null-frequency features through ablation experiments. The tenfold cross-validation technique and our own acquired 0-back working memory EEG signal data were selected for the validation assessment of STRGCN.

### Overall classification performance on dataset 1

**Fig. 1:**
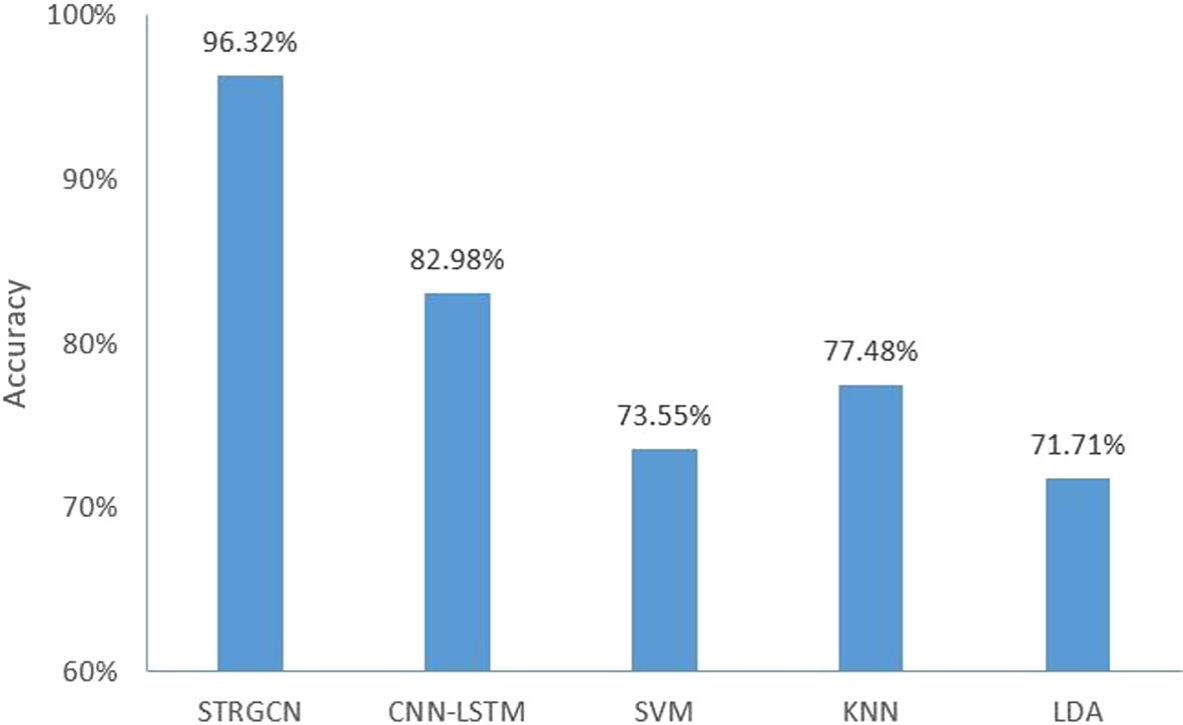
Classification results of classical classifier method and STRGCN on Zenodo

The accuracy of STRGCN on the test set is compared with the other four classification techniques, which exclusively employed PSD which is artificially extracted, and traditional classifiers which are SVM, KNN and LDA, as well as one deep learning methods, called CNN-LSTM. The results of the final comparison are shown in Fig. 1. From Fig. 1, it can be seen that KNN has the best classification results among the three classifiers with an accuracy 77.48%, while the other two classifiers only reach 73.55% and 71.71%.

**Fig. 2:**
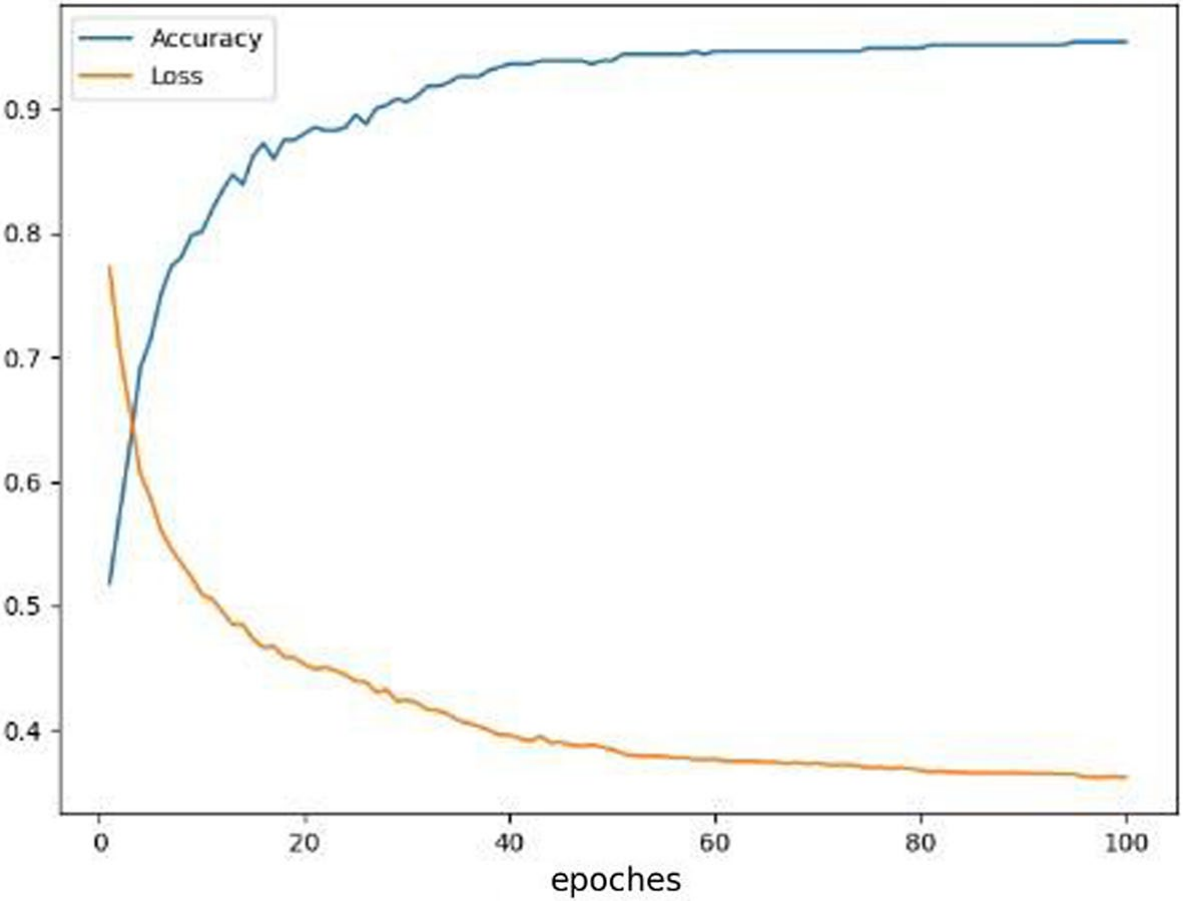
Accuracy and loss process for full band data with WC connectivity by the STRGCN model

**Fig. 3:**
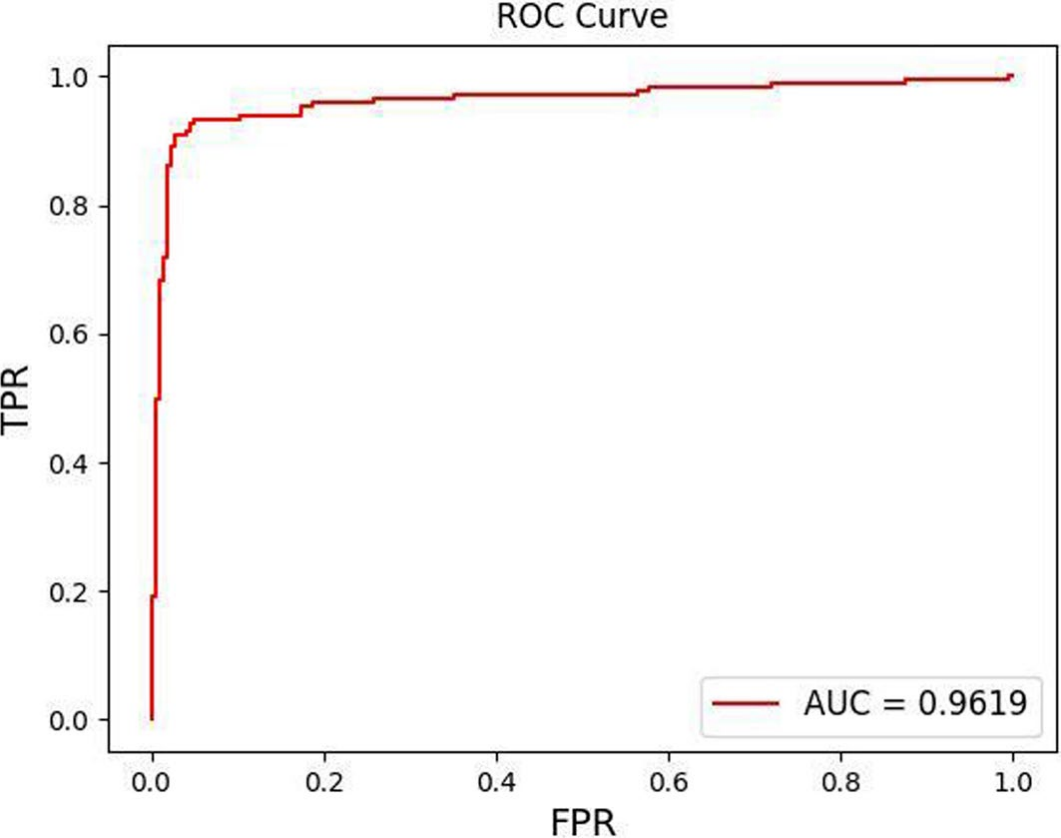
ROC curve for full band data with WC connectivity by the STRGCN model

The initial training period of the experiment was set to 200. In order to prevent overfitting problems caused by over-training, a validation-based early stopping strategy was used in the training, which made the number of final training cycles only a little over 100. Figure 2 shows the model improves in a relatively smooth training performance process, during which there is a clear trend of convergence in both accuracy and loss rate. Figure 3 indicates the stability and high performance of the proposed model on the testing dataset.

### Performance of ablation experiment

To evaluate the effectiveness of spatial graph convolutional layers and single-channel temporal convolutional layers in the model, two ablation experiments were conducted in this paper using temporal residual convolutional neural network (TRCN) and spatial residual graph convolutional neural network (SRGCN) notation, in which all four single-channel temporal convolutional layers were removed in SRGCN and two spatial graph convolutional layers were removed in TRCN. The two ablation models were compared with the full model, and the testing results are shown in Table 1.

**Table 1:** Classification results of ablation model and complete model

	Accuracy (%)	Recall (%)	Precision (%)	F1-score (%)
STRGCN	96.32	91.02	95.60	93.25
TRCN	73.47	59.17	74.07	65.79
SRGCN	87.76	72.62	98.39	83.56

### Crossover frequency experiment performance

**Fig. 4:**
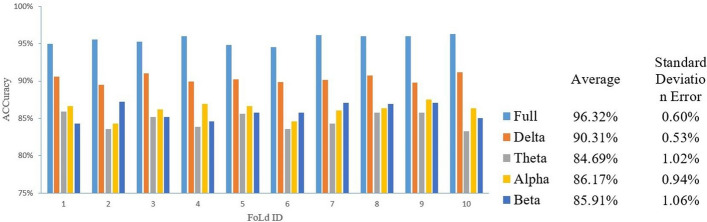
Classification results of tenfold cross-validation

In this experiment, we divided the full band into four sub-bands. They are delta (0.5–4 Hz), theta (4–8 Hz), alpha (8–13 Hz), and beta (13–30 Hz). Figure 4 shows the testing result of each band in each fold. The average classification accuracy of the full band is 96.32%, and sub-bands reached 90.31%, 84.69%, 86.17% and 85.91%.

### Performance of wavelet coherence as adjacency matrix

**Fig. 5:**
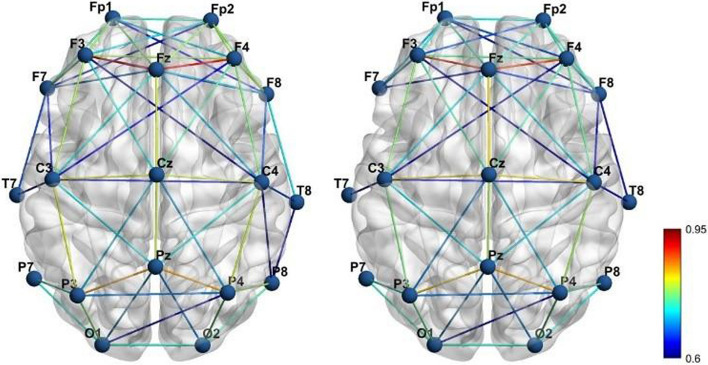
3D brain mapping used the averaged WC adjacency matrix, the left one is from HC and the right one is from SZ

To visually represent the connectivity between the channels of SZ and HC, we calculated the full-band average wavelet coherence coefficient matrices of the SZ and HC, respectively, and screened the channels with connectivity above 0.6. Figure 5 displays the 3D connectivity distributions of the SZ and HC brains obtained by the screening.

**Fig. 6:**
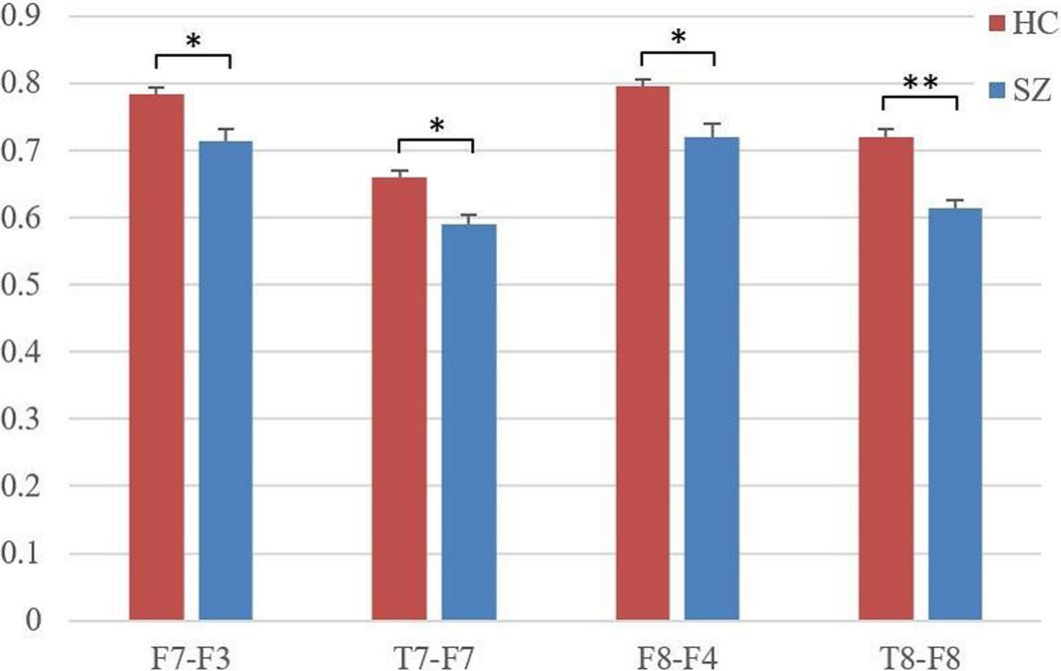
Significantly correlated groups of channels, with y-coordinate indicating the mean wavelet correlation coefficient, * indicating significant correlation and ** indicating extremely significant correlation in the figure

For the statistical study of the connectivity between each group of channels, we employed the independent sample t test method and used Bonferroni correction for correction. Finally, the channel groups with significant correlation (*p* < 0.05) and extremely significant correlation (*p* < 0.01) are summarized in Fig. 6.

### Classification performance on dataset 2

**Fig. 7:**
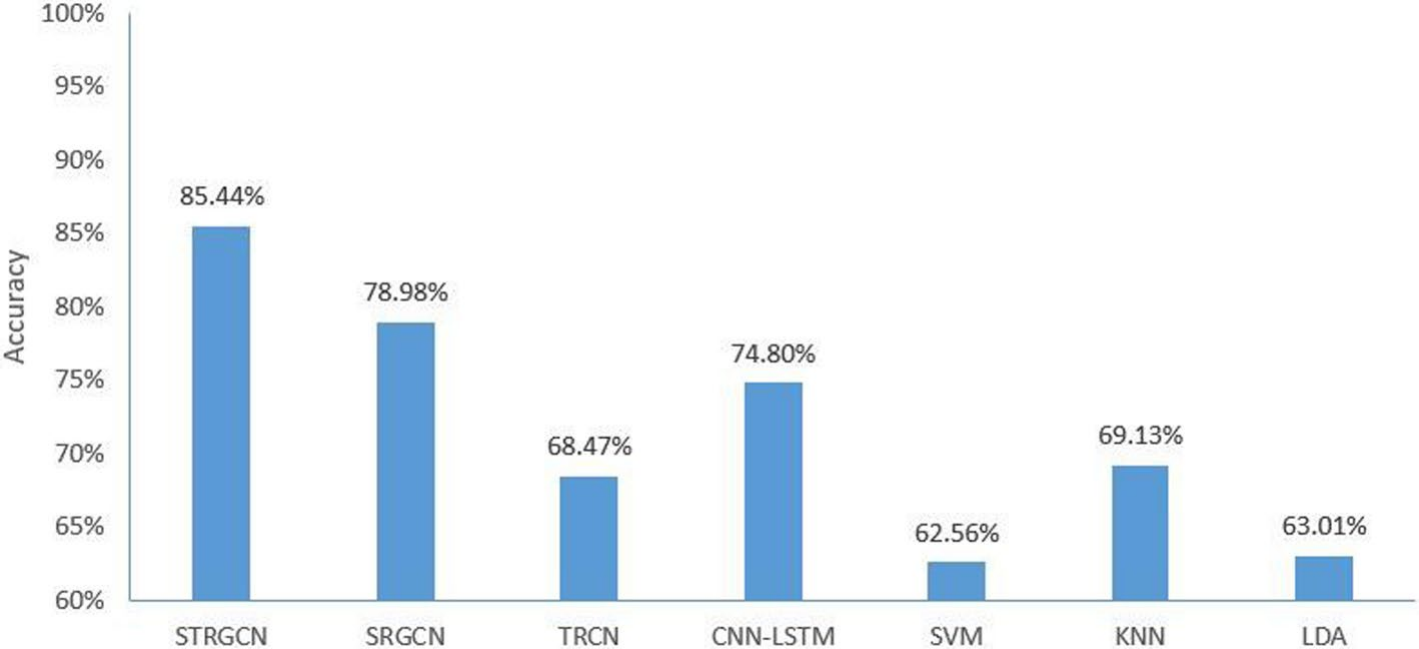
Classification results of STRGCN on dataset 2

To validate the generalization of STRGCN, we performed the same experiments using our own collected dataset, including comparisons with traditional machine learning and deep learning and ablation experiments, where the parameters used in all experiments were kept constant. The final testing results are shown in Fig. 7.

## Discussion

SVM, KNN and LDA are representations of traditional machine learning algorithms, and CNN-LSTM is a representation of traditional deep learning algorithms in the comparison tests. The results in Fig. 1 show that CNN-LSTM as a traditional deep learning model has a greater improvement in classification effect compared to traditional machine learning algorithms. However, all four algorithms are unable to fully analyze the implicit information of temporal and spatial frequency features contained in the EEG signal, the classification accuracy cannot reach the standard of spatial–temporal map convolutional neural network. The results in Fig. 1 also support this view.

It can be observed that STRGCN yields a substantial improvement in accuracy compared to traditional learning algorithms. The results in Fig. 2 demonstrate that the learning impact of STRGCN converges rapidly and stabilizes at a high level within the initial ten epochs. This indicates that STRGCN with both temporal and spatial frequency features has a significant advantage compared with traditional learning algorithms. To further validate this hypothesis, we created ablation experiments in which the STRGCN model is used separately to achieve the effect of shielding cross-nodes topologically associative features and node-specific sequential features on the classification results.

The findings in Table 1 further support the theory, showing that the complete STRGCN has a classification accuracy that is 22.85% and 8.56% higher than models in which spatial graph convolutional layers and single-channel temporal convolutional layers have been removed. It can be seen that both spatial graph convolutional layers and single-channel temporal convolutional layers can increase classification accuracy. The superiority of STRGCN performance is also demonstrated by comparing their F1 scores. Additionally, it was discovered through comparison that TRCN performs even worse than CNN-LSTM. This is due to the fact that TRCN only takes into account the time–frequency properties of the individual single channels' worth of EEG data. CNN-LSTM performs better than TRCN while still treating EEG data as ordinary data occurring in Euclidean space. This is probably because it evaluates the mutual effect across channels. This suggests that the temporal and spatial frequency features in the EEG signals can be extracted and analyzed simultaneously to obtain more EEG characteristics of SZ.

Each of five bands was used independently to classify the data using STRGCN method, and in tenfold cross-validation, the complete band consistently produced the best classification result. The best classification result among the four sub-bands belongs to delta, which is consistent with the findings of Bougou et al. [18].

It is clear from 3D brain topography that the functional brain connection between SZ and HC differs significantly. The wavelet coherence connectivity of HC is typically higher than that of SZ in the EEG data, as can be shown in Fig. 5, which displays the node connectivity over the threshold of 0.6. The frontal nodes and temporal lobes of the SZ, which have significantly weaker connectivity strength than the HC, are noteworthy regions. The node pairs with significant differences in Fig. 6 are also all in this region, providing strong support for the above conclusion. This region corresponds to the dorsolateral prefrontal cortex of the brain, which is a key area for executive functions. The reduced functional connectivity in this region may reflect the difficulties in cognitive control in SZ patients. This is consistent with the findings of Guo et al. [37].

The results of Figs. 4, 7 and Table 1 demonstrate the comparison results indicating whether in dataset 1 or dataset 2, the STRGCN method outperforms all other methods in terms of classification performance, and the traditional classifiers have the lowest results. This demonstrates the fact that STRGCN has good generalization. When we tried to alter the size of the convolutional kernel and step size in the temporal layer, we discovered that the classification performance of STRGCN altered very little. This phenomenon shows that the hyper-parameters of STRGCN have less of an impact and can be applied to real applications right away.

## Conclusions

To explore the differences in EEG signals between SZ and HC, we propose a spatial–temporal residual graph neural convolution network. This network sequentially extracts cross-channel topological correlation features and single-channel temporal causal features from EEG signals. It utilizes a graph convolutional neural network and a single-channel temporal convolution network, distinguishing it from the current mainstream CNN-LSTM model used in schizophrenia research and diagnosis.

The method collects spatial interaction information across EEG channels using brain networks that mirror the functional connectivity of the brain. It also mines single-channel dynamic temporal frequency information using single-channel temporal convolution centered on a causal network. The major purpose of this work is to confirm whether merging cross-channel topological correlation features as well as single-channel temporal variation features can disclose additional hidden information in EEG signals. Additionally, we aim to investigate the application of GCN-based methods. Unlike EEG-based brain network features or independent channel temporal features, we proposed STRGCN takes into account both spatial frequency and temporal frequency aspects within the EEG signal channel. This allows aberrant features in SZ patients to be detected in both the frequency response of localized regions and the functional connectivity across various regions.

In this study, we employed WC to determine the strength of the connections between the brain nodes. Compared with HC, we found that the connection strength of the SZ central and frontal regions is significantly reduced, and the connection strength between the two hemispheres is also substantially decreased. These phenomena can serve as a reference point for future research on the location of SZ lesions.

The research in this article still has certain limitations. Due to the small amount of data used, it needs to be further expanded to improve the learning ability of the model and reduce the data bias on the learning algorithm. In addition, although only one parameter, WC, is used in this article to calculate the connectivity between nodes, the parameters representing the connection strength between nodes can also be diversified. The sensitivity of different parameters to spatial features is also a feasible direction for future research.

## Methods

### Datasets

Dataset 1 in this paper were obtained from the open dataset Zenodo, which was recorded for 71 subjects (42 SZ patients vs. 29 healthy individuals) and included 32 channels of EEG data with 256 Hz sampling rate. The experiment created a task with a reward and punishment mechanism, where a monetary gain or loss set at $0.05 per trial. The task required that 4 simple shapes are presented to the subject 48 times in a pseudo-random manner (total number of trials 192), and the subject then needed to earn a reward (Win) or avoid a penalty (Avoid) by pressing a button (Go) or stopping their respond (NoGo). Therefore, the experiment will consist of 4 stimuli: Go-to-Win, Go-to-Avoid, NoGo-to-Win and NoGo-to-Avoid, where the probability of obtaining a reward or punishment for each stimulus was set at 80% and the subject is required to respond quickly to win more rewards.

At the beginning of the experiment, a crosshair appeared on the screen for 0.4–0.6 s. Then, the stimulus phase was commenced and the screen randomly presented a stimulus image staying for 1 s. After that, it entered a no-response period of 0.25-2 s followed by a response period of 2.5 s. Finally, the screen showed a feedback image after a 1-s cross and stayed for 2 s. According to the different stimuli above, the feedback types can be classified as positive, negative and neutral feedback. This paper only analyzed the potentials associated with events evoked by negative feedback, and selected 9 EEG channels (FP1, FP2, Fz, F3, F4, F7, T7, T8, C3, C4, Cz, Pz, P4, P3, F8, P8, P7, O2, O1).

All patients and healthy controls included in dataset 2 were recruited at Shanghai Yangpu Mental Health Center and all provided written informed consent. All SZ patients had their diagnosis confirmed over 1 month before recording their EEG while they had mild or moderate SZ based on their pre-scales examination. All patients were between 30 and 50 years old, including 36 SZ patients and 18 HC participants. EEG recording was undertaken with a 32-channel Neuroscan. EEG recording was performed during a simple working memory task. Each participant participated in a simple 0-back task. This task consisted of two major steps. At the beginning, the participant focused on the “+” symbol displayed on a white background. In the second step, a number between 0 and 9 was shown for 2 s on the screen. During this time, the participant needed to press the button on the keyboard to answer whether the number was 1. The program recorded the answer and send it to the experimenter.

### Dataset preprocessing

In this study, we collected EEG data from 36 SZ patients and 18 healthy individuals during the completion of the 0-back working memory task in order to validate the findings of the publicly available dataset, Zenodo [38]. We sampled the EEG signals at a sampling rate of 1024 Hz and selected a band-pass filter with a range of 0.5–30 Hz to remove external noise and the interference of the internal electrical signals, such as electrocardiogram. The EEG signal segments which are severely disturbed were then manually deleted for interpolation and filling, and independent component analysis (ICA) was used to remove the artifacts caused by head shaking and eye movement from the EEG signals.

### Wavelet coherence coefficient adjacency matrix

According to the research of Tafreshi et al., WC is generally recognized as a qualitative estimator that can depict the dynamic relationships between signals in the temporal frequency domain [39]. The definition of a wavelet transform is the convolution of an input value $x$ with a wavelet family $\psi \left(u\right)$:

1 $${W}_{x}\left(t,f\right)=\underset{-\infty }{\overset{+\infty }{\int }}x\left(u\right)\cdot {\psi }_{t,f}^{*}\left(u\right)du.$$

The wavelet transforms of input signals $x$ and $y$ can be used to calculate the wavelet cross-spectrum around time $t$ and frequency, i.e.,

2 $${CW}_{xy}\left(t,f\right)=\underset{t-\theta /2}{\overset{t+\theta /2}{\int }}{W}_{x}\left(\mu ,f\right)\cdot {W}_{y}^{*}\left(\mu ,f\right),$$

where $*$ defines the complex conjugate and $\theta$ is assumed as a frequency-depending time scalar. The formula for WC at time $t$ and frequency $f$ is described as:

3 $${WC}_{xy}\left(t,f\right)=\frac{|{CW}_{xy}\left(t,f\right)|}{{|{CW}_{xx}\left(t,f\right)\times {CW}_{yy}\left(t,f\right)|}^{1/2}}.$$

### Spatial–temporal residual graph convolutional neural network

Since the graph neural networks (GNN) have been proposed, they have been widely used in the characterization of non-Euclidean structured data [30]. Currently, there are two basic approaches to generalize convolution for structure graph data forms: spectral-based GNN and spatial-based GNN. In the region of spectral-based GCN research, Bruna et al. presented the first prominent model by applying convolutions in spectral domains with graph Fourier transforms [36]. Since then, there has been an increase in research on improving and extending spectrum-based GNN [40, 41]. Spatial-based GNNs define graph convolutions by rearranging vertices into certain grid forms which can be processed by normal convolutional operations [42, 43].

In the field of graph processing, GNNs can embed complex network structures into meaningful low-dimensional representation features [44]. Two-dimensional convolution is the process of taking the pixel values of the nodes within a certain range adjacent to each node and performing a weighted average. In two-dimensional image processing, each pixel point of the image can be regarded as a node whose pixel values are obtained by filtering its surrounding pixel points through a specific filter. The process of weighted average the information of the vicinity of each node when generalizing the information of the graph from two-dimensional to high-dimensional is known as graph convolution. The neighborhoods of high-dimensional nodes will be more complicated and disorganized than those of two dimensions.

**Fig. 8:**
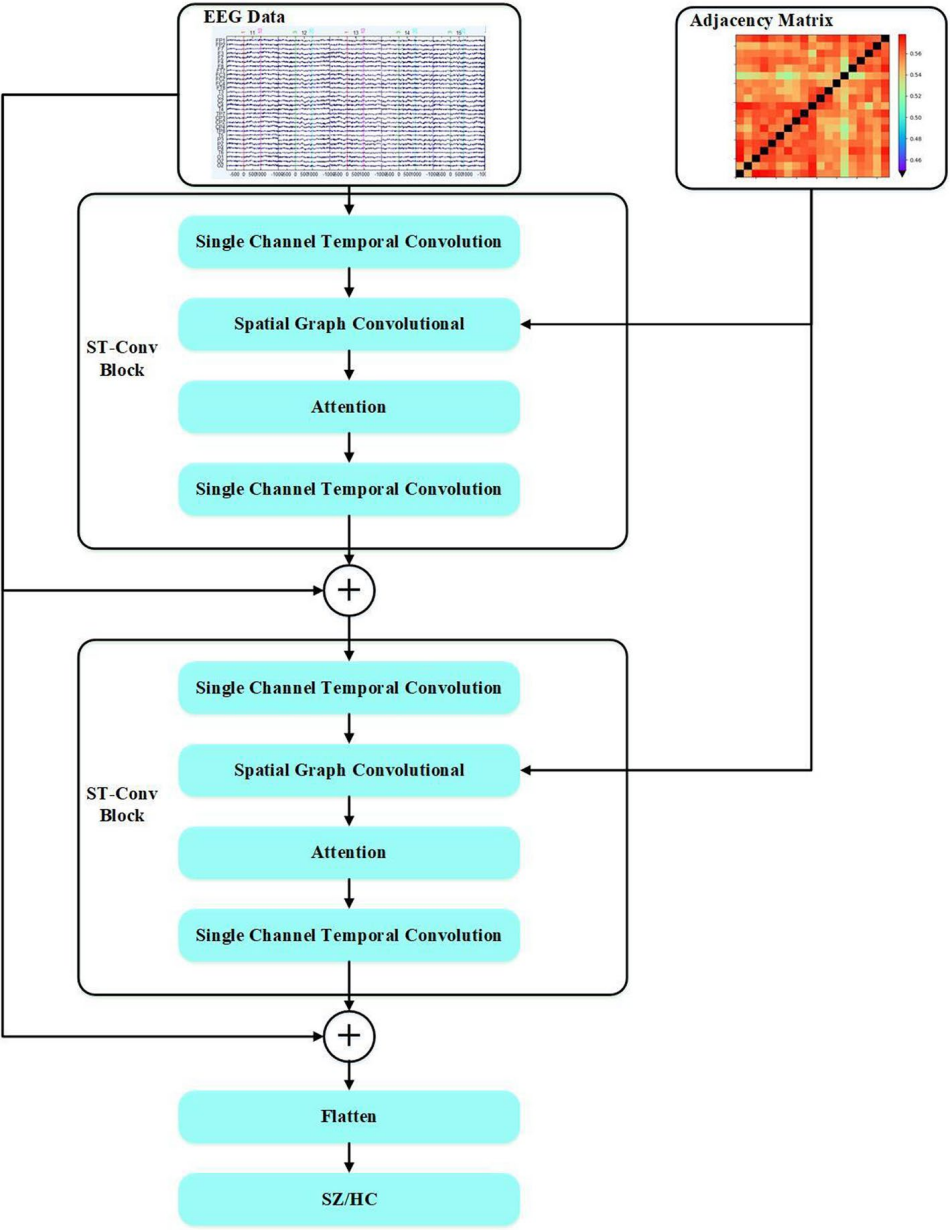
An illustration of spatial–temporal residual graph convolutional neural network

As shown in Fig. 8, the proposed STRGCN model as a whole consists of two spatial–temporal convolutional (ST-Conv) blocks. Each ST-Conv block consists of a single-channel temporal convolutional layer, a spatial graph convolutional layer, an attention layer, and another single-channel temporal convolutional layer in that order. Layer normalization is used in each spatial–temporal convolutional block to prevent overfitting. The single-channel temporal convolutional layer extracts time–frequency features from the single EEG channel. The spatial graph convolutional layer combines the EEG channels and their adjacency matrix in a unified process to extract the correlation features between the time domain and the spatial domain. The attention layer enables the model to focus on the channels that are more affected by the disease. Residual learning is performed after each ST-Conv block to alleviate the problem of gradient dispersion or gradient explosion cause by the increasing depth of the deep neural network. The final classification result is obtained by integrating all features in each final flatten layer.

#### Spatial graph convolutional layer

We introduce the notion of a graph convolution operator $*\mathcal{G}$ multiplying a signal $x\in {R}^{n}$ in the spatial space with a kernel $\Theta$:

4 $$\Theta *\mathcal{G} x=\Theta \left(L\right)x=\Theta \left(U\Lambda {U}^{T}\right)x=U\Theta \left(\Lambda \right){U}^{T}x,$$

where the graph Fourier basis $U\in {R}^{n\times n}$ is a matrix of eigenvectors of the normalized graph Laplacian $L={l}_{n}-{D}^{-\frac{1}{2}}W{D}^{-\frac{1}{2}}=U\Lambda {U}^{T}$, ${l}_{n}$ is an identity matrix, $D\in {R}^{n\times n}$ is the diagonal degree matrix with ${D}_{ii}=\sum_{j=0}^{n}{W}_{ij}$, $\Lambda \in {R}^{n\times n}$ is the diagonal matrix of eigenvalues of $L$.

The signal sequence $x$ is filtered through the kernel $\Theta$ by means of Eq. (4). However, due to its $\mathcal{O}\left({n}^{2}\right)$ complex multiplications, the use of Chebyshev polynomials as well as first-order approximation is considered to simplify the calculation where $\Theta$ is simplified with respect to the polynomial $\Theta \Lambda = \mathop \sum \limits_{k = 0}^{K - 1} \theta_{k} \Lambda^{k}$, where ${\theta }_{k}$ is a vector of polynomial coefficients, $K$ is the kernel size determining the maximum radius of the convolution from central nodes. When $\Lambda$ is rescaled as $2\Lambda /{\lambda }_{max}-{l}_{n}$, where ${\lambda }_{max}$ denotes the largest eigenvalue of $L$, Chebyshev polynomial ${T}_{k}(x)$ is traditionally used to approximate kernels as $\Theta \Lambda \approx \mathop \sum \limits_{k = 0}^{K - 1} \theta_{k} T_{k} \left( \Lambda \right)$. Then the graph convolution in Eq. (4) can be rewritten as:

5 $$\Theta *\mathcal{G} x=\Theta \left(L\right)x\approx \sum_{k=0}^{K-1}{\theta }_{k}{T}_{k}\left(L\right)x,$$

where ${T}_{k}(L)\in {R}^{n\times n}$ is the Chebyshev polynomial of order $K$ evaluated at the scaled Laplacian $L=2L/{\lambda }_{max}-{l}_{n}$. In this way the amount of computation can be reduced to $\mathcal{O}(K\left|\varepsilon \right|)$, further assuming that ${\lambda }_{max}$ can be taken approximately to 2. Thus $2L/{\lambda }_{max}-{l}_{n}\approx L-{l}_{n}$=$-{D}^{-\frac{1}{2}}W{D}^\frac{1}{2}$, and Eq. (5) can be simplified to

6 $$\Theta *\mathcal{G} x\approx {\theta }_{0}x-{\theta }_{1}\left({D}^{-\frac{1}{2}}W{D}^{-\frac{1}{2}}\right)x.$$

In order to reduce the number of parameters involved in the calculations, ${\theta }_{0}$ and ${\theta }_{1}$ are replaced by a single parameter by letting ${\theta }_{0}={-\theta }_{1}=\theta$. By renormalizing $\widetilde{W}=W+{l}_{n}$ and $\widetilde{{D}_{ii}}=\sum_{j=0}^{n}\widetilde{{W}_{ij}}$. Then the graph convolution operation for a one-dimensional signal can finally be expressed as:

7 $$\Theta *\mathcal{G} x=\theta \left({l}_{n}+{D}^{-\frac{1}{2}}W{D}^{-\frac{1}{2}}\right)x=\theta \left(\widetilde{{D}^{-\frac{1}{2}}}\widetilde{W}\widetilde{{D}^{-\frac{1}{2}}}\right)x.$$

Extending this idea to signals with ${C}_{i}$ channels, the graph convolution can be generalized to

8 $$\Theta {y}_{j}=\sum_{i=0}^{{C}_{i}}{\theta }_{i,j}(L){x}_{i},$$

where $1\le j\le {C}_{0}$ and ${C}_{0}$ represents the number of output channels, Thus there are a total of ${C}_{0}\times {C}_{i}$ Chebyshev coefficients to be determined.

#### Single-channel temporal convolutional layer

**Fig. 9:**
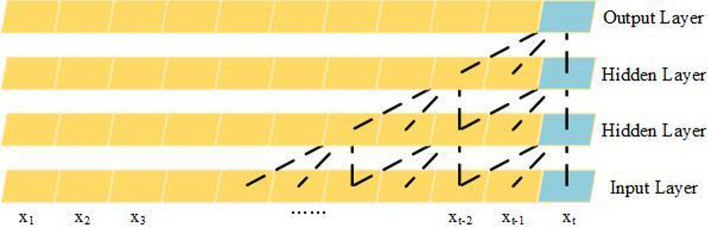
Structure of the causal convolutional model

We employ an entire convolutional structure on a temporal axis to capture sequential dynamic behavior of EEG recordings as Gehring et al. have demonstrated that CNNs have the superiority of fast training in sequential-series analysis [45].We adopt a causal convolution-based method to extract features from the time series, as each EEG signal is only influenced by the current and previous brain activity. As shown in Fig. 9, each convolutional layer contains a 1D convolution with a kernel, followed by a rectified linear unit (ReLu) function as a nonlinearity. Additionally, the convolution only utilizes data collected at that time and before. Taking ${X}_{t}$ as an example, the value of ${X}_{t}$ in each layer is convolved with ${X}_{t}$, ${X}_{t-1}$ and ${X}_{t-2}$ of the previous layer. After the $i$ th layer, the output ${X}_{t}$ contains the features of {${X}_{t-i-1},{X}_{t-i}\cdots \cdots {X}_{t-1},{X}_{t}$} of the input layer, and the closer the temporal distance from ${X}_{t}$, the more influence it has on the output ${X}_{t}$. The width and step size of the kernel can be set according to the depth of the layer.

An input of a sequential convolution for each node can be regarded as a length-M sequence with ${C}_{i}$ channels. By applying the same convolution kernel to each channel node in graph equally, the temporal convolution can also be generalized to the entire graph.

#### Attention layer

By taking advantage of the link between features across channels, we create a channel attention layer. The layer aims to highlight the important areas of the input image, as each channel of the feature map is thought of as a feature detector [46]. We reduce the spatial dimension of feature map in order to compute the channel attention effectively. Average-pooling has so far been widely used for aggregating spatial data. However, we argue that max-pooling acquires yet another crucial piece of information about distinctive object properties to infer finer channel-wise attention [47].

**Fig. 10:**
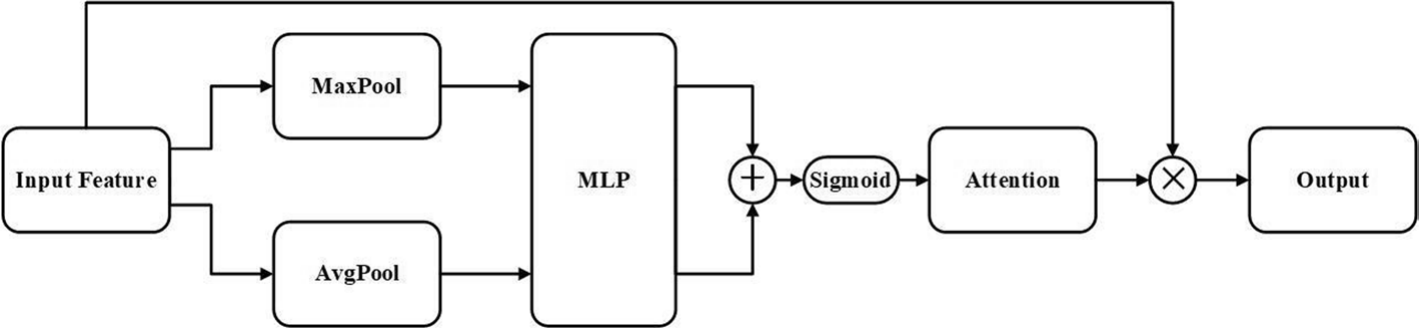
Structure of the attention layer in ST-Conv

The main structure of attention layer is shown in Fig. 10. Initially, we aggregate the spatial information of a feature map by using both average-pooling and max-pooling operations, generating two different spatial context descriptors. Subsequently, a shared network receives both descriptors to create our channel attention map. Multi-layer perceptrons (MLP) with one hidden layer make up the shared network. Element-wise summing is used to combine the output feature vectors after the shared network has been applied to each descriptor. To summarize, the channel attention is computed as follows:

9 $${\text{M}}_{c}\left(F\right)=\sigma \left(MLP\left(AvgPool\left(F\right)\right)\right)+\text{MLP}\left(MaxPool\left(F\right)\right),$$

where $\sigma$ denotes the sigmoid function. At last, the output is obtained by multiplying the input features with the channel attention.

#### Residual layer

Simply increasing the learning depth for a basic network model might result in gradient dispersion or gradient explosion, and the typical method for this issue is to regularize the layers in order to regularize the feature matrix. The training set loss will increase as the network depth is increased, but it will reduce as the number of network layers in the model increases because as the number of network layers rises, the training set loss steadily declines and eventual stabilization. Assuring consistency of inputs and outputs, adding a residual block between two ST-Conv blocks provides the network with the ability to transfer information across layers, highlights minute variations by including a direct mapping. By doing so, the model is able to disregard from the same body parts throughout each cycle.

### Performance evaluation metrics

In order to assess the performance of the classification methods, accuracy, recall, precision and F1-score were chosen to evaluate the model performance in this paper. They are represented using Eqs. (10), (11), (12), (13), respectively, where $\text{TP}$ stands for true positive, $\text{TN}$ stands for true negative, $\text{FP}$ stands for false positive and $\text{FN}$ stands for false negative:

10 $$\text{Accuracy}=\frac{\text{TP}+\text{TN}}{\text{TP}+\text{TN}+\text{FP}+\text{FN}},$$

11 $$\text{Recall}=\frac{\text{TP}}{\text{TP}+\text{FN}},$$

12 $$\text{Precision}=\frac{\text{TP}}{\text{TP}+\text{FP}},$$

13 $$F1=\frac{2\times \text{TP}}{2\times \text{TP}+\text{FP}+\text{FN}}.$$

### Cross validation

Cross validation is a validation technique used to evaluate the generalizability of results. In prediction problems, it is common to first train the model using a training dataset and then test it using a dataset that is completely independent of the training dataset to evaluate the performance of the model in actual operation. Among these methods, k-fold cross-validation is a commonly used technique.

In k-fold cross-validation, the original data set is randomly divided into k equal-sized subsamples. Among k subsamples, one subsample is selected as test data at a time, and the remaining k-1 subsamples are used as training data. This process is repeated k times, ensuring that each subsample is used as test data once. Ultimately, these k results can be averaged to get a single estimate of model performance. A significant advantage of this approach is that all data are used for training and validation, and each data is used only once for validation, thus providing a stable and reliable assessment of model performance.

## Data Availability

The data of Zenodo can be obtained from 10.5281/zenodo.29601 and 10.5281/zenodo.29064.
